# Expression of Concern: An integrated view on society readiness and initial reaction to COVID–19: A study across European countries

**DOI:** 10.1371/journal.pone.0294386

**Published:** 2023-11-09

**Authors:** The PLOS ONE Editors

*PLOS ONE* conducted a post-publication assessment of this article [1] due to questions about aspects of the peer review process. In the post-publication assessment, which involved a member of *PLOS ONE*’s Editorial Board and an external reviewer, concerns were raised about the rigor and appropriateness of the statistical analyses and whether the article’s conclusions are well-supported by the data.

This research was conducted in the first year of the global COVID pandemic. This does not alter the journal’s requirements with regard to research reporting standards and publication criteria, but in this context and given the objectives of the study the *PLOS ONE* Editors understand that there were constraints which may reasonably have impacted study design decisions. The authors commented that when the article was written, “many insights [had] yet to be developed and data were scarce. This fundamentally influenced our conceptual framework and the chosen methodological approach.”

Even so, based on the outcome of the post-publication assessment, *PLOS ONE* has concerns about the robustness of the results and the strength of evidence supporting the article’s conclusions due to the study design limitations. Specific concerns include: (a) limited variables were used to represent readiness structures, responses, and outcomes, (b) the data analysis approach did not include a robust statistical analysis, and (c) potential biases in the dataset and study design were not adequately addressed or discussed in the article [1].

Overall, the outcome of the assessment was that the results reported in the original published article did not adequately support the conclusions. The *PLOS ONE* Editors issue this Expression of Concern to notify readers of these issues and additional information and analyses that were provided in post-publication discussions.

As is discussed below and in S1 File, the authors provided additional context, clarifications, and analyses in post-publication discussions that partially resolved the concerns and lend additional support for the article’s main claims. With this provided, the journal considers that this body of work makes a valid contribution to the published literature, although not all concerns have been fully resolved.

Primary data underlying the study’s results and other supplementary materials (referred to below as “OSF Supp. Materials”) are available at https://osf.io/g69hy/?view_only=9c54713bd17c4dac8c8c1a6104d67d45.

Below, we outline the main concerns raised in this case and the authors’ response to each. For additional information, see S1 File.

1. The conceptual framework is limited in its scope of what determines society readiness and reactions. The article did not adequately address the rationale for aspects of the study design, or provide evidence to support that the included factors provide a reasonable, reliable representation of the context. For example, concerns were raised about the scientific validity and appropriateness of representing health risk factors by a singular variable (the percentage of population aged 65 or more), and why health risk of age was used as an indicator of society readiness for a public health emergency.
  With regard to health risk factors, the authors commented that when the study was conducted–during early stages of the pandemic–unambiguous research and data on risk factors were limited, the authors viewed age as the only reliable indicator known at that time. The article’s conclusions (final paragraph of [1] discuss this limitation and note that the choice of dimensions should be revisited in the future; for example, future studies could combine characteristics of population (age, gender, obesity) and medical conditions (prevalence of diabetes, cardiovascular diseases, etc.).
  The authors also explained that use of age as a health risk indicator in the study was in line with the conceptual framework of the study: societal readiness seen as demographic predisposition for a more severe outcome of the pandemics and societal reaction in terms of occurrence of government interventions targeting elderly population.
2. The statistical analyses presented in the article are not suitable to address the aims of this study, or for use in analyzing outcomes that represent a cumulative distribution function. The analyses did not adequately address key confounds, such as under detection of COVID cases, different case definitions, and the timing of epidemic waves and first reports of COVID cases within the country being analyzed. Also, the study did not include sensitivity analyses, and Tables 3 and 5 did not present analysis of variance across the countries in a transparent way. Due to the lack of statistical rigor applied in these data analyses, the claims reported in the article based on the correlation analysis are not adequately supported.
  The authors explained that their approach to this study was not rooted in statistical modeling or an approach where independent variables are evaluated to see how they affect outcomes. Instead, the study was designed based on an approach similar to theory-based evaluation which strives to systematically link concept (dimensions within proposed conceptual framework) and data (indicators derived in operationalization phase). This conceptual analytical model relies on so called ’empirical indicants’; a small subset of possible indicators used to represent a particular concept [2]. The authors further noted that the results are not dependent on or sensitive to the order to dimension inclusions, and that the correlation analysis (Table 4) should be understood only as a “showcase performed to initially check possible redundancy between variables” i.e. Table 4 is not intended to demonstrate relationships between variables based on statistical modeling analyses.
  The authors also provided the following clarifications about the analysis method used in the study:
  - ELECTRE MLO was used as an analytical support because it has been shown to be effective for evaluation of multidimensional phenomena and as an alternative to common composite indexes.
  - This approach is suitable for mix-data type and has meaningful interpretation of performance differences, and ELECTRE based analysis have been previously applied in epidemic and pandemic research [3–6]).
  - The method was used to demonstrate the usability of proposed framework, and to obtain relative positioning of countries according to the depicted dimensions of readiness and reaction. Only basic elements of ELECTRE-MLO were used due to constraints of the study design (e.g. variables used, data quality, time frame). The outcome reflected in three color-coded groups of countries was used to monitor movements in hierarchy obtained by outranking method and was not intended to offer a “forecasting tool in terms of the initial severity of the COVID-19”.
3. The article did not adequately address biases in the study design and/or analyses, discuss what steps were taken to address, minimize, or otherwise mitigate effects of bias, or to discuss impacts of potential biases as limitations of the study. For example, the article does not address how global or regional responses might have affected country-level responses, preparedness, or outcomes; the data collected for each country may have been biased due to the relative timings within the study period of within-country COVID exposures and waves of positivity and COVID-related mortality; and issues pertaining to under-detection and differences in case definition need to be addressed in analyses to examine the reliability and robustness of the results.
  The authors commented that per the reported analysis countries’ responses were not uniform despite the global context and information available about responses in other countries. Some governments started with early measures well before their first case. For example, Croatia, Bulgaria, and Norway introduced measures (GovI1) as early as the end of January, whereas others did not take action until their first cases were reported. In an effort to account for differences in country-level exposure timings, the authors defined three sub-indicators related to government measures: timeliness, strictness, and duration of government interventions. The authors noted that although COVID deaths are a lagging indicator, since all countries from the sample were at the peak of infection at a similar time (March-April), death counts declined during May (OSF Supp. materials/Examples/picture 4.).
  Country-level COVID timelines are in OSF Supp. materials/Timeline.
4. Apple mobility data do not provide a consistent representation of population-level mobility across countries due to differences in iPhone usage. The authors noted that data about iPhone users in the sample range from the lowest in Poland (5% of all mobile phone users i.e. approx. 1.5–2 million users) and Serbia (10% of all mobile phone users i.e. approx. 400.000–500.000 users) to the highest Sweden (50% of all mobile phone users) and UK (50% of all mobile phone users) [OSF Supp. Materials/Apple mobility trends]. They conducted a comparison of Google and Apple tracking data for Poland and Serbia (countries with the lowest portion of iPhone users in the sample) and found a correlation of ~94% (OSF Supp. materials/Examples/figure 6.)). These data should be interpreted with caution since Apple and Google use different methodologies to track movement, but this finding supports that users of iPhones vs. other devices may have comparable mobility patterns.
5. The study’s main conclusions reported in the Abstract and the ‘Discussion and conclusions’ section of [1] overstate what can be drawn from this study’s data. The penultimate sentence of the Abstract (“Our findings confirm…”) is to be disregarded. The ‘Discussion and conclusions’ section of the article is hereby updated with the following new text, which replaces paragraph 2 (with all three bullet points) and paragraphs 6 to 10 (from “If we recall our correlation analysis…” to the paragraph ending with reference 44).

## Discussion and conclusions

Bringing together the diverse aspects which shape society readiness, reaction and outcomes for the COVID-19 pandemic is a challenging research task as many relevant aspects are not yet understood. Following the course of theory-based evaluation we have systematically derived an integrated approach to study preparedness and initial reaction to COVID-19. Based on the body of knowledge and available data in the first phase of the outbreak (from January to May of 2020) we have extracted in total six dimensions, of which three were related to society preparedness and another three to initial reaction to the pandemics. We have proposed a way to operationalize these dimensions by extracting corresponding indicators and conducting a pilot exercise using a sample of 23 European countries. It is important to stress that the proposed approach produced only one view on the initial phase of the COVID-19 outbreak across the observed European countries. Due to many limitations associated with available data and body of knowledge in the early phase of the pandemic outbreak, conclusions of this study should be taken with caution. As the body of knowledge on COVID-19 is continuously growing, but still not released of ambiguity, it is reasonable to say that findings themselves are more in the domain of discussion points than strongly supported standpoints.

The conclusions are built around the finding that in the first phase of the COVID-19 outbreak, society reaction could compensate for its lower preparedness to pandemics. There are some indications that this pattern of reaction was more associated with Eastern European countries in the early days of COVID-19 outbreak. For updated discussion on possible reasons for differences in reactions to the COVID-19 outbreak between Eastern and Western European countries please see S1 File.

Although our six-dimensional framework is not designed to be a forecasting tool in terms of the expected severity of the COVID-19, the chosen structural and process dimensions gave a fair reflection of the actual outcome in the first phase of the outbreak. Still, it should not be seen as a rigid structure. The major challenge is about the operationalisation of the proposed conceptual framework–choosing adequate indicators for each dimension. Even though, we selected indicators based on the results of systematic search, the need to reconcile drawn dimensions with available indicators and data unavoidably simplified the concept. Limitations of the research are outlined further below and in S1 File.

Regarding indicators and data used in this study it is important to emphasize that they were collected in the early stage of the pandemics which brings several limitations of the study. The first one is about the outcome data (morbidity and mortality) used to classify countries in three color coded groups (red, green and yellow, Table 6). As noted in several studies [7, 8], countries use incompatible methodologies for counting infected and deceased patients, which makes comparing data problematic. Similar to other early COVID-19 studies we relied on data from official national sources, but their validity may be questioned by the fact that they were unadjusted and many countries later updated numbers on cases and deaths as reporting caught up or case definitions were amended. As highlighted by WHO [9], excess mortality, even though not unambiguous, is a more objective and comparable measure that accounts for both the direct and indirect impacts of the pandemic (undetected cases, lives lost due the overwhelm of health care system, etc). But, in early stage of COVID-19 outbreak these data records were lagging even more. WHO published first estimates on May 20^th^ and they are still being updated. However, our follow-up analysis based on excess mortality showed that grouping of countries from our sample (yellow, green and red) was not disturbed comparing to unadjusted data (please see S1 File for further elaboration).

Another limitation also coming from scarce data in early COVID-19 analysis is related to the absence of adjusting caseloads to testing ratios. True infection rates remain questionable, since data based on the number of positive tests per day was to some extent influenced by a country’s willingness and ability to undertake testing. This calls for adjusting caseloads to testing ratios. As we have already explained, many of the data at that time were not yet available or valid for all countries (such as prevalence studies for antibodies) and official data without any adjustments were often used in the early scholar papers. We have however acknowledged the importance of testing by including it as one dimension in our framework. Still, this remains the strong limitation of our study and with new and more reliable records being developed (such as prevalence studies for antibodies), another direction for improving outcome indicator is adjusting caseloads to testing ratio.

Another limitation that can be associated with our study refers to the timeline of the outbreak. In an infectious disease where death lags behind cases, the amount of time where vulnerable (nonimmune populations) are exposed to a disease is of great importance. Using the same length of time (16 weeks, Feb—May 2020) for all countries in this study did not significantly impact the outcomes and findings. The peak of infections and deaths in the first wave of pandemics in Europe had occurred during April, so by the end of May, all countries in the sample were at the end of the first wave of epidemic were at the peak of infection at a similar time (see S1 file). Using the same length of time for all countries also brings to discussion the impact of global or regional responses on country-level reaction and outcomes. As we discussed above we were aware that the reaction of the countries could not be observed individually, i.e. that it was related to cases in other countries and that it affected the reaction of the countries. Global response was not fully included in our analysis since it is still a very debatable issue, even labelled as a “silent factor of COVID-19” [10]. For example, as elaborated in [11] several studies on COVID-19 concluded that domestic factors strongly shape policymaking, even when faced with a global threat and when problem pressure is high. Another study indicated that from a comparative perspective, managing the COVID-19 crisis in Europe has been predominantly shaped by distinct country-specific approaches [12]. Despite the complexity and unambiguity, global response remains an important aspect which should be accounted for in further research on holistic frameworks of society response to COVID-19.

To sum up, the availability of data and confirmed factors strongly influenced the operationalization of our proposed framework. However, this study offers a valuable platform that is flexible for further development and adaptations. As new relevant factors are unambiguously confirmed and data records become more reliable the choice of indicators should be enriched in future applications. The choice of dimensions should also be revisited in the future. Pandemics are complex situations and it takes time to collect all the pieces of the puzzle, especially if the analysis is to be expanded to other regions and a larger set of countries.

6. The article cited a publication (Reference 32) that was retracted prior to the publication of the *PLOS ONE* article. The sentence that cites this reference (“Moreover, the increased risk of hospital death was over the age of 65 (mortality of 10.0%, vs. 4.9% among those aged ≤65) and current smoking [32].”) is supported by other published works including articles cited in the original article [13,14] and [15–17].

## Supporting information

S1 File Supplementary discussion. Additional discussion of points raised in the post-publication assessment.
(DOCX)
