# Supplementary material for: An integrated view on society readiness and initial reaction to COVID–19: A study across European countries
Source: PLoS One. 2020 Nov 23;15(11):e0242838. doi: 10.1371/journal.pone.0242838 (PMC7682891; doi:10.1371/journal.pone.0242838)
Supplement: Attachment — Submitted filename: Response to Reviewers.docx [file pone.0242838.s001.docx]

***Response to Editor***

## We have now prepared a revision of the manuscript entitled " An integrated view on society readiness and initial reaction to COVID–19: a study across European countries," Ref. No PONE-D-20-27597.

Together with the revised manuscript (marked copy and unmarked version), we send responses to reviewers' comments. Revision files have been prepared in accordance with the requirements of the PLOS ONE's style.

The answer to your remark “*Please upload a copy of Figure 4, to which you refer in your text on page 25. If the figure is no longer to be included as part of the submission please remove all reference to it within the text.*” is: The text refers to Figure 3. We have corrected this typo in the revised manuscript.

Thank you for your time and consideration.

We look forward to your reply.

Sincerely,

Authors

***Response to Reviewer 1***

Thank you for your helpful and constructive comments. Please find our answers listed below.

***R1#:*** *I commend the efforts of the authors in contributing to literature on pandemics, especially COVID-19. However, I do have some concerns with some constructions of sentences. More editing and proofreading is needed, emphasising on punctuations.*

**Answer:** Thank you for careful reading and constructive suggestions. We have rephrased unclear parts of sentences and text. Also, we corrected all typos, and the language is improved and cross-checked again. All changes are visible in the file “Revised Manuscript with Track Changes“. Corrections to the parts you referred to are listed point-by-point in the table below.

| ***R1#:*** | **Answer:** |
| --- | --- |
| ***Introduction:*** | |
| *'This allowed us to more sophisticatedly analyse how European countries stand relative to each other in term of readiness and reaction to COVID-19'. Why is the word sophiscatedly' used in this sentence? I suggest 'to rigorously analyse'* | We have used "rigorously analyse" instead of "more sophisticatedly". Thank you for the suggestion. |
| *'The second is whether the application of more rigorous measures can complement to the preparedness capacity of a country. Please delete 'to' in this sentence.* | It is deleted. |
| ***Materials and Methods:*** | |
| *'Namely this gave us the chance to bring the early outcome of pandemics (in terms of morbidity and mortality) together with readiness structure and reaction process'. What are you 'namely' here? I don't understand this sentence construction.* | "Namely" is excluded and the whole sentence is rephrased to make it more clear and now is as follows: *This gave us the opportunity to connect the early outcome of the pandemic (in terms of morbidity and mortality) together with readiness structure and reaction process.* |
| *'With the Covid-19 outbreak one of the key question was will well prepared health systems be resilient against the COVID-19 epidemic' Is this a statement or a question?* | It is a statement. We have rephrased this sentence. Now it is…*The outbreak of the COVID-19 called in question the ability of well-prepared health systems to respond to the epidemic* |
| *'Beside preparedness and trust one more important element of country reediness'. Readiness and not reediness. Please correct* | It is corrected. Thank you. |
| *'Eventually, we encompass all dimensions and hypothesise that the obtained performance levels correspond the most to the actual outcome'. Plural od hypohtesis is hypotheses. Please correct.* | In this sentence “hypothesise” was used as a verb. To avoid further confusion we have changed this sentence and now it is as follows: *Eventually, we include all six dimensions and analyse to what extent obtained performance levels correspond to the actual outcome.* |
| ***Results:*** | |
| *'Low performance by the same criterion pushed Netherland and Germany down'. Netherlands* | It is corrected. Thank you. |
| ***Discussion and conclusions:*** | |
| *'Our findings revile whole complexity of efforts to put Covid-19 outbreak under control'. Please check; revealed and not reviled, which means criticizing abusively or angrily.* | We have changed it to ‘*revealed’*. Thank you |
| *'Unlike western European countries...' Should be Western.* | We have corrected this. |
| *'However, a better overall preparedness score for the Western health care systems does not necessarily mean that they are better equipped for fighting against viruses such a Covid-19.' Should read 'such as Covid-19.* | We have changed it to *‘such as’.* |
| *'It is important to remind that this data reading is set around statistics on the initial outbreak and a number of countries are yet to reach the peak of their outbreaks'. 'to remember...'* | We have used *‘to remember’* instead of *‘to remind’*. Please note that this part of the manuscript is additionally changed according to the remarks from the Reviewer #2. |

Regarding your question on data availability, we would like to point out that all data were collected from publicly available sources (e-SPAR database, Our World in Data, OxCGRT Tracker, Apple Community Tracker, and European Value Study). Along with the raw data for each variable, these sources are presented in the last column of the Table 2, and included in the list of references (with link to the source). We have added a source for testing policy data (in Table 2 and the reference list) which was omitted in the original submission.

*Thank you very much once again for your help.*

***Response to Reviewer 2.***

Thank you for the comments that guided us to improve our paper. Please note that our answer has two parts, the first refers to the presentation of the methodology and the second concerns additional referencing.

**R#2:**   *Job well-done author but the methodology used was not clearly specific and outlined, pls outline the study methodology process. For example study designs, duration, population, eligibility, source, search strategy.* *Pls mention the sampling strategy e.t.c*

Answer: To clearly show our methodological approach and make it easier to follow, we have restructured section *Materials and methods* of our manuscript. It now has two subsections, one on the conceptual framework and the other on the methodological basis of our study. To outline the methodology of our study, we have included a step-by-step description of the methodology process at the beginning of subsection *Methodological approach*. Each methodological step is further explained in terms of design, data source, time-frame for data collection, etc in separate subsections (*Operationalisation of dimensions*; *Sampling and data*, *Correlation among dimensions* and *A method for cross-country analysis*).

Regarding the sample for cross-country analysis in our study, our aim was to include approximately the same number of Eastern and Western European countries so that we could investigate (di)similarities between them. The final set was conditioned by the availability of data. After examining the data sources (for the established indicators and variables), we were able to perform our analysis for 23 European countries (12 Western European and 11 Eastern European). Please note that a separate subsection (*Sampling and data)* is added to explain our sampling strategy.

**R#2:** ….*and additional referencing to support some of your claim in the study, especially from line 530- 543. Thank you!*

**Answer:** Following both your and Reviewer 3 suggestions, we have included additional references in support of our claims, provided below. The part you referred to (on expectations regarding country reopening and relaxing the measures) has been revised, and several recent reports and studies have been added to ground the statements. This part now reads as follows:

*However, for economic reasons, lockdown is not a long-term solution. Several studies evidenced that European countries will face socio-economic losses due to COVID-19 epidemics [85-6] and that the consequences will be different in Eastern and Western European countries [87]. Hence, the Eastern European countries have yet to see the seriousness of COVID-19, as measures are relaxed and the capacity of the health system loses support in lockdowns and bans [88]. This is already obvious based on data from the end of July 2020, which show that COVID-19 is again very active in Serbia and some other neighbouring countries [89].*

*On the other hand, the Western European countries, although not rigid in the first response, will probably cope better with COVID-19 in the future. It is important to remember that this reading of the data is set around statistics on the initial outbreak and that a number of countries have yet to reach the peak of their outbreaks, as implied in reports from October 2020 [90].*

New references are:

[20] Tangcharoensathien V, Calleja N, Nguyen T, Purnat T, D’Agostino M, Garcia-Saiso S, Landry M, Rashidian A, Hamilton C, AbdAllah A, Ghiga I. Framework for managing the COVID-19 infodemic: methods and results of an online, crowdsourced WHO technical consultation. Journal of medical Internet research. 2020;22(6):e19659

[59] OWiD (2020), Our world in Data database, Available at: <https://ourworldindata.org/grapher/tests-per-confirmed-case-daily-smoothed?time=latest>

[61] Kandel N, Chungong S, Omaar A, Xing J. Health security capacities in the context of COVID-19 outbreak: an analysis of International Health Regulations annual report data from 182 countries. The Lancet. 2020 Mar 18.

[85] Kraus S, Clauss T, Breier M, Gast J, Zardini A, Tiberius V. The economics of COVID-19: initial empirical evidence on how family firms in five European countries cope with the corona crisis. International Journal of Entrepreneurial Behavior & Research. 2020 May 26.

[86] Lenzen, M., Li, M., Malik, A., Pomponi, F., Sun, Y-Y., Wiedmann, T., et al. (2020). Global socio-economic losses and environmental gains from the Coronavirus pandemic. *PLoS ONE* 15(7): e0235654. <https://doi.org/10.1371/journal.pone.0235654>

[87] Avrămescu, T. C. (2020). Assessments on the Effects of the Coronavirus Pandemic on the Economies of the Central and South-East European Union Countries. *Ovidius University Annals, Economic Sciences Series*, *20*(1), 105-112.

[88] Loblova, O., Borbath, E., Rone, J., (September 18, 2020). Coronavirus: why central and eastern European countries seem to be running out of luck, *The Conversation*, Retrieved from: <https://theconversation.com/coronavirus-why-central-and-eastern-european-countries-seem-to-be-running-out-of-luck-146349>

[89] Associated Press (July, 12, 2020). COVID-19: Surge in new coronavirus cases in Eastern Europe prompts new restrictions, *Euro News*, Retrieved from: <https://www.euronews.com/2020/07/12/covid-19-surge-in-new-coronavirus-cases-in-eastern-europe-prompts-new-restrictions>

[90] Sangameswaran, S., Ahluwalia, S. (October, 9, 2020). Europe records 100,000 daily coronavirus cases for first time, *Reuters*, Retrieved from: <https://www.reuters.com/article/us-health-coronavirus-europe-cases-idUSKBN26U1Q0>

*Thank you for your time and help.*

***Response to Reviewer 3.***

Thank you for the comments and guidelines to improve the manuscript. Find our answers listed below.

**R#3*:***   *It is well written manuscript. A few grammatical errors were detected and a thorough review of sentences is suggested.*

**Answer:** We have corrected all typos in the revised manuscript, and the grammar has been improved throughout the text. All changes are visible in the “Revised Manuscript with Track Changes“.

**R#3:**   A few relevant research articles are suggested that can further supplement the introduction and discussion section. They are as follows:

Kandel N, Chungong S, Omaar A, Xing J. Health security capacities in the context of COVID-19 outbreak: an analysis of International Health Regulations annual report data from 182 countries. The Lancet. 2020 Mar 18.

Tangcharoensathien V, Calleja N, Nguyen T, Purnat T, D’Agostino M, Garcia-Saiso S, Landry M, Rashidian A, Hamilton C, AbdAllah A, Ghiga I. Framework for managing the COVID-19 infodemic: methods and results of an online, crowdsourced WHO technical consultation. Journal of medical Internet research. 2020;22(6):e19659.

Kraus S, Clauss T, Breier M, Gast J, Zardini A, Tiberius V. The economics of COVID-19: initial empirical evidence on how family firms in five European countries cope with the corona crisis. International Journal of Entrepreneurial Behavior & Research. 2020 May 26.

**Answer:** Thank you for pointing out the additional references that have enriched our manuscript. In addition to suggested ones, we have included several others to support some of the claims in the manuscript (as suggested by the Reviewer#2). They are:

[59] OWiD (2020), Our world in Data database, Available at: <https://ourworldindata.org/grapher/tests-per-confirmed-case-daily-smoothed?time=latest>

[86] Lenzen, M., Li, M., Malik, A., Pomponi, F., Sun, Y-Y., Wiedmann, T., et al. (2020). Global socio-economic losses and environmental gains from the Coronavirus pandemic. *PLoS ONE* 15(7): e0235654. <https://doi.org/10.1371/journal.pone.0235654>

[87] Avrămescu, T. C. (2020). Assessments on the Effects of the Coronavirus Pandemic on the Economies of the Central and South-East European Union Countries. *Ovidius University Annals, Economic Sciences Series*, *20*(1), 105-112.

[88] Loblova, O., Borbath, E., Rone, J., (September 18, 2020). Coronavirus: why central and eastern European countries seem to be running out of luck, *The Conversation*, Retrieved from: <https://theconversation.com/coronavirus-why-central-and-eastern-european-countries-seem-to-be-running-out-of-luck-146349>

[89] Associated Press (July, 12, 2020). COVID-19: Surge in new coronavirus cases in Eastern Europe prompts new restrictions, *Euro News*, Retrieved from: <https://www.euronews.com/2020/07/12/covid-19-surge-in-new-coronavirus-cases-in-eastern-europe-prompts-new-restrictions>

[90] Sangameswaran, S., Ahluwalia, S. (October, 9, 2020). Europe records 100,000 daily coronavirus cases for first time, *Reuters*, Retrieved from: <https://www.reuters.com/article/us-health-coronavirus-europe-cases-idUSKBN26U1Q0>

*Thank you for your time and help.*
