# Supplementary material for: Expression of Concern: An integrated view on society readiness and initial reaction to COVID–19: A study across European countries
Source: PLoS One. 2023 Nov 9;18(11):e0294386. doi: 10.1371/journal.pone.0294386 (PMC10635477; doi:10.1371/journal.pone.0294386)
Supplement: S1 File — Additional discussion of points raised in the post-publication assessment. (DOCX) [file pone.0294386.s001.docx]

**Overview comments by the article’s authors**

This paper is not only about the early phase of pandemics but it was also written during the early research phase of the pandemic when many insights are yet to be developed. By conducting thorough review of the literature at that time we were able to reveal that the prevailing stream of research is based on statistical investigation of specific, mostly medical, factors with the aim to detect their causalities with the outcome of COVID-19. Much less attention was paid to reflect the multidimensional nature and complexity of COVID-19 related phenomenon, where our study is accommodated. One of the main challenges and the contribution of our work was to set a framework that brings together institutional, societal and medical aspects while characterizing them by their nature (readiness/structure and reaction/process). Such framework provides a structure within which to present the aspects needed to enable feedback to policy makers which was the substantial aim of our study.

The paper is primarily based on a conceptual analytical approach; statistical tools were not in our focus. The ELECTRE MLO was used as an analytical support because it is proven to be very effective for evaluation of multidimensional phenomena and as an alternative to common composite indexes. Besides, it is suitable for mix-data type and has meaningful interpretation of performance differences.

**Supplementary materials and primary data**

Supplementary materials and primary data for the study are available at: <https://osf.io/g69hy/?view_only=9c54713bd17c4dac8c8c1a6104d67d45>

The Supplementary materials include a timeline in the form of a table and time charts for each country from the sample. The table provides dates of: the first case, the first government intervention, peek of government interventions and critical mobility reduction. The time charts further illustrate the dynamics of these variables in the first three months of pandemic. The charts are organized in separate folders according to Fig 2. right (structure and reaction dimensions) and are discussed in following answers.

The Supplementary materials also include a Table that can be used as a legend for flags, abbreviations, and country-level information (please see Supp. materials/Country legend).

**Additional comments on specific issues raised in the post-publication assessment**

*The choice of included and excluded factors*

During the work on our conceptual framework we examined additional dimensions that were not discussed in the paper.

In developing our conceptual framework, we did not rely on statistical modelling based on random testing of possible indicators. The approach used is more in line with TBE (Theory based evaluation) i.e. a conceptual analytical model which strives to systematically link concept (dimensions extracted based on SPO in or paper) and data (operationalization of dimensions in our paper). In such research setting indicators (also labelled as empirical indicants) are used to approximate concept empirically. As well explained by Zeller&Carmines^[[1]](#footnote-1)^ “*indicants are never able to fully exhaust nor completely duplicate the meaning of theoretical concepts. Any particular set of empirical indicants that one chooses, therefore, is only a small subset of an almost infinite number of possible indicants that could be selected to represent a particular concept*.”

Now, we are coming to the question of indicators and variables which depicted our set of six dimensions. First it should be kept in mind that during April and May 2020 when this framework was built, the unambiguous research findings as well as international data sources were scarce. This is the reason why for example Health risk was portrayed only by age as, in our opinion, the only reliable indicator at that moment.

*Were the included factors, untransformed, the best representation of the context?*

At the time of our analysis cross-national studies on COVID-19 were still in infancy but already pointing out to the need to scrutinize even widely recognized preparedness metrics. For example Aitken et al. (2020) (ref [6] in the paper) analysed 1000 countries and founded that countries with higher GHSI (Global Health Security Index) did not have higher COVID-19 rate. The later paper (published after ours) confirmed this finding.^[[2]](#footnote-2)^ Moreover, other metrics that can be associated with country’s capacity to deal with the spread of infectious decease like IDVI and INFORM (end its follow up ERI) are built up around the concept of vulnerability while WHO’s SPAR database and GESI are more oriented towards preparedness and refer to the countries that make up the States Parties to the IHR International Health Regulations. We decided on WHO’s SPAR because at the time it was found to be acknowledged as metrics of preparedness functional capacity (references [7] and [8] in the paper) and up to our knowledge not criticized as GHSI. As we stated in our paper ‘several studies dealing with preparedness and vulnerability in the context of COVID-19 have emphasised the importance of social factors [ref. [6](https://journals.plos.org/plosone/article?id=10.1371/journal.pone.0242838#pone.0242838.ref006)–[8](https://journals.plos.org/plosone/article?id=10.1371/journal.pone.0242838#pone.0242838.ref008) in the paper]. It is true that we did not go into deep analysis of this aggregated value but we did acknowledge that differences are not large among countries (Table 5 in Supp. materials- indicator 1, mean score is 7.74 with moderate level of dispersion at 1.05). As we highlighted in the paper the emerging criticism around preparedness metrics and call for including social aspects invoked us to complement functional capacity (health system and its preparedness for a health crisis) with two more dimensions trust and risk factors.

*Rationale for using age 65+ as a health risk factor, and variation in this indicator among the countries studied*

The age variation among the countries was addressed by Bauer et al. (2020)^[[3]](#footnote-3)^, where they examined age dependence of COVID-19 deaths independently in first and second half of 2020 in 12 European countries overlapping with our study. They included individuals of ≥ 40 years old, this covered 98.3% of the registered COVID-19 deaths. The lowest COVID-19 mortality risk was estimated for Norway, followed by Denmark and Germany with mortality rates of 0.004% (95% confidence interval, 0.003–0.005), 0.009% (0.007–0.012), and 0.013% (0.011–0.015), respectively. The larger values were observed in England and Wales (0.092%; 0.086–0.098), Belgium (0.090%; 0.080–0.101), and Scotland (0.085%; 0.074–0.098). A remarkably wide range of the estimates of age dependency for COVID-19 mortality was noted with the largest risk ratio observed in Switzerland (5.66; 5.36–6.00). The risk ratios for Belgium (3.98; 3.70–4.28), and Austria (4.07; 3.85–4.31) were closest to the median of the 16 countries. Roughly, in terms of the median over countries, the risk of COVID-19 mortality in 2020 for an increase in age of 10 years increased by a factor of around 4. Differences in age dependency of COVID-19 mortality between the first and second half of the year were found among the 15 countries. For some countries, age dependency was more accentuated in the second half of the year and was more attenuated in the first half for others. This is consistent with the reported analysis of data in 11 overlapping European countries, where the age distribution of COVID-19 deaths was compared between the two waves in 2020 and only small fluctuations were reported^[[4]](#footnote-4)^.

The cut off at 65 for age was used as this is a common demographic cut-off for elderly populations, and has been associated with marked difference in IFR estimates between European countries^[[5]](#footnote-5)^. Older adults are more likely to get severely ill from COVID-19. More than 81% of COVID-19 deaths occur among people over age 65. The number of deaths among people over age 65 is 80 times higher than the number of deaths among people aged 18-29^[[6]](#footnote-6)^. On April 6, 2020, the report systematically reviewed COVID-19-related mortality data from 13 European countries. The summary proportions of persons < 40, 40-69, and ≥ 70 years of age among all COVID-19-related deaths were 0.1% (0.0-0.2%; I2 24%), 12.8% (10.3-15.6%; I2 94%), and 84.8% (81.3-88.1%; I2 96%), respectively. People under 40 years of age represent a small fraction of the total number of COVID-19-related deaths in Europe^[[7]](#footnote-7)^. It is true that majority of COVID-19 deaths happened ≥ 80 years of age: England 57.5%, Italy 59.4%, Germany 65%, Sweden 66.6%, Switzerland 69.9%, in accordance to reports until the beginning of November 2021^[[8]](#footnote-8)^, but it is still largely lower comparing to ≥70 years of age^[[9]](#footnote-9)^

*Potential impacts of other health factors, and robustness of the results to indicator selection*

As was discussed above, this study was not based on statistical modelling which would entail testing independent predictors for cumulative outcome. Instead, it was based on theory based evaluation which strives to systematically link concept (dimensions within our conceptual framework) and data (indicators derived in operationalization phase). In such research setting indicators are used to approximate concepts empirically.

In line with that we extracted the age of population because it was singled out at the time. As highlighted in a report by the Norwegian Institute of Public Health at the time: *“We conclude that age stands out as the predominant single risk factor for severe disease”* or in Nature *“The studies reveal that age is by far the strongest predictor of an infected person’s risk of dying — a metric known as the infection fatality ratio (IFR), which is the proportion of people infected with the virus, including those who didn’t get tested or show symptoms, who will die as a result.”^[[10]](#footnote-10)^* What also drew us towards this indicator is its alignment with our conceptual framework (societal readiness) - demographic predisposition for a more severe outcome of the pandemics and societal reaction in terms of occurrence of government interventions targeting elderly population.

Additional evidence that emerged in COVID-19 research after this study has indicated that the health risks dimension would be enriched by combining characteristics of population (age, gender, obesity) and medical conditions (prevalence of diabetes, cardiovascular diseases, etc.).

*Differences in timing of country-level COVID infection peaks may have influenced the results, given that COVID deaths are a lagging indicator and the study period was a fixed 16-week period.*

The concern is certainly reasonable, but the data show that this issue can be to a large degree neutralized. Namely, it is true that some Western European countries (that have gone through the worst), had their first case at week 5 - 6 of 2020 comparing to those (that had better outcomes on average) which had their first case of COVID-19 notified in week 10, but also data show the following: all “red” countries which had their first case in the fifth or sixth week had accumulated only a negligible number of cases by the end of the week 9 (on average well below 1% of all cases). For example, until the 1/3/2020, four weeks after the 1^st^ case, Spain accumulated only 45 cases and no deaths of 239479 total cases and 27127 total deaths by and of May 2020; UK accumulated 61 cases and no deaths of 257539 total cases and 37527 total deaths; Germany accumulated 79 cases and no deaths of 183410 total cases and 8540 total deaths; Sweden accumulated 14 cases and no deaths of 38661 total cases and 4395 total deaths; even Italy with accumulated 1128 cases and 29 deaths was below 0.5% of total cases (232997) and total deaths (33415). The peak of infections and deaths in the first wave of pandemics in Europe had occurred during April, so by the end of May, all countries in our sample were at the end of the first wave of epidemic. This can clearly be seen from graphs in the Supp. materials/Examples/figures 2 and 4.

Therefore, although it is true that COVID deaths are a lagging indicator, since all countries from the sample were at the peak of infection at a similar time (March-April) death counts considerably have declined during the May (Supp. materials/Examples/figures 4 and 5).

Similarly, as regard the inadequate levels of testing we believe that early time of the first case does not automatically lead to a bad assessment of the testing indicator (number of days with the number of tests below 10 per case), but it was a matter of crisis management. Especially in light of the fact that there were few cases in the first days, so there was no need to conduct a large number of tests according to the parameter that we included. Thus, countries like Germany or Finland, although they had the first case notified early, have the best scores according to this parameter. On the other hand, the Netherlands or Serbia, even though they were among those who had the first case notified later, were among the worst rated.

Based on the excess mortality data we conclude that the cumulative number of deaths and infections is not biased to the extent that it would rearrange grouping of countries in green, yellow and red. Countries that are marked as red in our sample, i.e. those that had the most severe outcome in the first 4 months of the pandemic, are also countries that have the highest excess mortality compared to green and yellow, with excess mortality around or below zero (Supp. materials/Examples 1a and 1b).

*Rationale and suitability of correlation analyses for studying outcomes that represent a cumulative distribution function*

This work was not built on approach where independent variables are randomly included to see how it affects the outcome. Cumulative cases and mortality have only been used to simultaneously monitor and compare the performance obtained by the ELECTRE MLO (expected performance) with the actual outcome (achieved performance) of the pandemic in first four months as stated in the paper. The only reason why we marked countries with colours (based on cumulative cases and mortality) was to look at our results (relation trees in Figures 1-3) through the lenses of the severity of pandemics. To sum up, the chosen method (ELECTRE MLO) was not used for censored dataset and cumulative outcome, but to obtain relative positioning of countries according to the depicted dimensions of readiness and reaction. To be even more precise Tables 3 and 5 used to build Fig.1, Fig.2 and Fig.3 do not include data on cumulative outcome (Table 6). Accordingly, the method was not chosen on the basis of suitability for cumulative data but as a validated approach for cross-country analysis when multidimensional phenomenon is researched and different data types used.

Furthermore, cumulative outcomes are not used in the correlation table (Table 4). Why and how the correlations are presented is explained in the answer to Academic Editor. We agree that cumulative outcomes should be further scrutinized if they are to be used in the framework of statistical analysis. To remove the bias in terms of how they were used here we have provided analysis on the basis of excess mortality (discussed below).

The correlation analysis should be understood only as a simple showcase, performed to initially check possible redundancy between variables of which some have been created by authors (three for GI and two for MR). However, bearing in mind the aim of the study as well as the sample size and reliability of data we did not build up the methodology on the line of statistical methods and therefore the related text was not part of the result section. When it comes to the statement that MR is a direct consequence of GI, it should be seen as a sum up of two previous sentences which point to specific variables (GovI1, MobR2 and Gov3 and Mob3). We have now provided illustrations of this relationship using time charts for each country from which is obvious that stringency (GovI1, GovI2, GovI3) and mobility (MobR1, MobR2) are directly related (please see Supp. materials/Timeline).

Conclusions about causality cannot be drawn from the observed correlations, but since we know that government interventions were introduced to reduce peoples’ mobility it is to be expected that mobility was influenced by government restriction and not another way around.

The outcome reflected in three colour-codded groups of countries was used to monitor movements in hierarchy obtained by ELECTRE-MLO and with no intention to offer a “forecasting tool in terms of the initial severity of the COVID-19” (as stated in the paper). Namely dimensions (and associated indicators and variables) on one side and cumulative morbidity and mortality on the other are not used as independent and dependent variables as explained above.

We have added data on excess mortality for countries in the sample. They imply that in the period analysed in this paper the excess mortality would not change the grouping of countries (yellow, green and red) based on cumulative cases and deaths (please see Supp. materials/Examples 1a and 1b).

*Previous use and/or validation of* *ELECTRE MLO in studies of pandemic response or for studies involving multiple contexts*

As an outranking approach, ELECTRE method has already found its applications in the epidemic and pandemic field of research (e.g. ELECTRE III^[[11]](#footnote-11)^ ELECTRE-MOr^^[[12]](#footnote-12)^^ ^[[13]](#footnote-13)^, ELECTRE-VICOR combination^^[[14]](#footnote-14)^^). In this paper ELECTRE-MLO was chosen because it is suitable for mix-data type, and the authors have extensive experience with using it to capture multidimensionality of the phenomena in various fields of application (ICT policy, education, economics and logistics).^[[15]](#footnote-15)^ The most recent application is for benchmarking the European transnational Innovation policies^[[16]](#footnote-16)^

Having constraints coming from the nature of indicators (variables, quality of data) and limited time frame (initial phase of the outbreak) we decided to use only basic elements of ELECTRE-MLO which offers hierarchical representation of relative performance instead of strict ranking of evaluation units. In the future research it can be applied in its full potential, i.e. as a decision making tool in the benchmarking framework. The main analysis and derived conclusions is about figure 3 where we demonstrated how including reaction dimensions is repositioning the countries. The cut-offs for green/red /yellow countries could be defined with another mortality and morbidity rates, but the vertical mobility of the countries (Figure 3) would not change. Also, the results are not dependant on the order of dimension inclusions.

*Robustness of the results to the limitation of COVID underdetection*

We considered data for the excess mortality and concluded that the grouping countries in green, yellow and red (based on outcome severity) is not disturbed (please see Supp. materials/examples/files 1a and 1b). Countries that are marked as red in our sample, i.e. those that had the most severe outcome in the first 4 months of the pandemic, are also countries that have the highest excess mortality compared to green and yellow, which have excess mortality around or below zero (see Supp. materials/Examples 1a and 1b). In addition, other studies based on excess mortality analyses confirmed this. For example in the Report OECD, & European Union Health at a Glance: Europe 2020^[[17]](#footnote-17)^ it is clearly stated: “Nevertheless, over the first ten months of 2020, data from reported COVID‑19 and excess mortality rates suggest Belgium, Italy, Spain and the United Kingdom were the most severely affected, followed by France, the Netherlands and Sweden. In contrast, most countries in Central and Eastern and South-eastern Europe, as well as most Nordic countries, have been less adversely affected by the first wave of the pandemic.” Please note that the conclusion from this report is related to first ten months, while we have analysed first four months of pandemic.

*Validity or rationale for treating of each country's response as independent instead of incorporating or discussing impacts of global or regional responses and country-level timings*

Global and regional response during the early stage of pandemic is highly complex issue and couldn’t have been addressed in details in our paper. We agree that the global context was important and that countries have looked to each other to some extent. The note of a global response was made in the first part of our paper where we stated: “*It is fair to say that the rapid and strong reaction of the Chinese government and its success in fighting the virus in February 2020 influenced many other countries in terms of their strategies related to the outbreak of COVID-19. What has happened in China has shown that quarantine, social distancing, and isolation of the infected population can contain an epidemic* [Ref. [25](https://journals.plos.org/plosone/article?id=10.1371/journal.pone.0242838#pone.0242838.ref025) in the paper].”

Yet, our analysis shows that despite that, the countries' responses were not uniform. Some governments started with early measures well before their first case. By the time when many of the East European countries had their first case, they were not looking at a new disease. At the beginning of the March Italy diagnosed nearly 6,000 cases in a week and the few days later, on the March 11 the WHO had declared a global pandemic. Baring that in mind it is fair to say that important part of a success for Eastern European countries in the reaction to Covid19 can be assigned to latecomer’s advantage. Yet, it should be noted that some governments started with early measures well before their first case (Croatia, Bulgaria and Norway) while many others waited until the first cases were recorded, regardless of the situation in other countries. This is a complex issue and there is evidence that countries did not responded uniformly due to many interplaying factors. Although rapid and strong reaction of the Chinese government and its success in fighting the virus in February 2020 may encouraged some countries to implement stringent measures, it remains unclear to what extent this was emulated on the regional or global level.^[[18]](#footnote-18)^ Studies are still very careful in conclusions about patterns of reaction, like for example the existence of East–West divide in response to COVID-19^[[19]](#footnote-19)^. Some authors claim that international coordination was largely absent, since international organizations such as the European Union and WHO played a very modest role in coordinating immediate national responses.^[[20]](#footnote-20)^

Leaving aside the timings of reaction it is possible that some of the reasons for the different reaction to initial outbreak of Covid-19 can be attributed to socio-political differences among Eastern and Western European countries. For example, our data showed ([Table 3](https://journals.plos.org/plosone/article?id=10.1371/journal.pone.0242838#pone-0242838-t003)) that countries with less severe initial outbreaks, i.e. Easter European countries, had an average trust score of 2.6 (on a four step reverse scale) compared with a score 2.06 in countries with a more severe initial outbreak. This can lead to explanation, which is in the line with some other studies ^[[21]](#footnote-21)^ ^[[22]](#footnote-22)^. that less trustworthy governments had to count on quick lockdown interventions instead of relying on the conscience of citizens’ (to follow recommendations). This in turn, could lead to a better result compared to more trustworthy countries.^[[23]](#footnote-23)^ However, although low social trust could play a positive role in the initial months of the pandemic, it is unclear whether this will continue to be the case as the current situation evolves. Low social trust can also lead to catastrophic effects when countries reopening is characterized by uncertainty and lack of clear direction, as manifested in the US and some Balkan countries during the summer of 2020. Furthermore, countries with much longer democratic tradition might have more difficulty taking forceful of even appropriate action comparing to former communist countries especially those with populist governments.^[[24]](#footnote-24)^

When it comes to our indicators related to government interventions we are aware that (as for measuring the speed of reaction) those who had their first case early and those who had it later were not in the same position, but that is why we defined three sub-indicators related to government measures – *timeliness*, *strictness* and *duration* of government interventions. Our goal was to account for these differences. Namely, if some countries did not react quickly enough, they had the opportunity to react with stricter measures and for those strict measures to last longer.

Furthermore, 9 out of 23 countries reduce the movement below 40% (our benchmark for mobility reduction) between March 14 and 16 (Austria, France, Spain and the majority of Eastern European countries). However, Germany and England, which were among the first to have the first cases, as well as Switzerland and the Netherlands, recorded a mobility reduction 7-15 days later, as a result of a “slower tightening” of measures. In line, we may question why these countries, which had their first case at the same time with Croatia and Romania, did so much worse than them?

If we leave aside the first case and just look at the dates of reaching highest stringency values we may see that Italy achieved the stringency of 90 (100 is max) on March 20, Spain 85 (March 30), Netherlands 79 on March 31, while on the other hand Serbia on March 21 reached maximum possible stringency index of 100, followed by Croatia who reached 96.3 on March 23. We may wonder what forced the Serbs and Croats to introduce such a strong measures much earlier than Spain or the Netherlands, even though they all participated together in the same global context? Why Spain or the Netherlands, Germany, Finland, Sweden had not been scared of the huge death or other factors that the Academic Editor cites, and Serbia Bulgaria Croatia are? We have tried to explain this with awareness of weaknesses, but of course there may be some other explanations that are equally relevant. This is certainly a discussion point which in early days of COVID-19 was circulated in media (reference 78 from the paper and The Guardian^[[25]](#footnote-25)^), but also in some scholarly papers published at the time^[[26]](#footnote-26)^ ^[[27]](#footnote-27)^ ^[[28]](#footnote-28)^ ^[[29]](#footnote-29)^).

*Concern about lack of support for claims that the Eastern European countries may have performed better because they had better hospital facilities*

This was not our conclusion but a discussion of the possible causes for better performance of the Eastern countries. It was a speculation about the results and that is why we have used the word “may” to separate it from the positive statements. We have also mentioned some other possibilities from the literature clearly indicating that they fall into the category of speculations: “There is some speculation about the protection from BCG vaccine that is still mandatory in Eastern European countries [Ref. 83 in the paper].”

Our intention was to tackle possible explanations that should be explored in the future research and that is why only provided some first insights about hospital capacities. Before the sentence quoted by the Academic Editor we included some indicative findings from the literature: “For example, when it comes to the number of hospital beds per 100,000 inhabitants in 2018, ex-communist countries occupied 8 of the first 10 and 11 from the first 15 positions in Europe [Ref. 78 in the paper]. This is important because around 1/5 of COVID-19 patients who have moderate or serious illness require hospitalization [Ref. 79, 80 in the paper]. Among hospitalized patients with COVID-19, the percentage of patients who required ICU care has varied from 5% to 10% in Europe [28, 81].” It all should be only understood as hinting possible causes for deep elaboration which would certainly go beyond simple number of beds or IVU units as the Academic Editor pointed out.

*Country-level differences and proportions of the populations represented in the Apple mobility indicator data*

The data about iPhone users in our sample range from the lowest in Poland (5% of all mobile phone users i.e. approx. 1.5-2 million users) and in Serbia (10% of all mobile phone users i.e. approx. 400.000-500.000 users) to the highest Sweden (50% of all mobile phone users) and UK (50% of all mobile phone users). The number of people covered by Apple trackers in our sample far exceeds even the largest samples in social research, so there seems to be no doubt that the Apple mobility indicator represented the true mobility in countries. Furthermore, there is no indication, to our knowledge, that, in average, iPhone users would demonstrate different patterns of movement comparing to Android (mobile phone) users. To remove doubts for the purpose of comparability, in the Supp. materials (example 6) we give a comparison of Google and Apple trackers for Poland and Serbia (countries with the lowest portion of iPhone users in the sample). In this example is shown that the correlation between Apple and Google trackers is about 94%, almost at the theoretical maximum, although it should be kept in mind that Apple and Google use different methodologies to track movement, (we have compared Apple’s stream ‘walking’ and Google’s data on ‘transit stations’). Finally, many COVID-19 studies in Europe have used Apple mobility data.^[[30]](#footnote-30)^ ^[[31]](#footnote-31)^^[[32]](#footnote-32)^

1. Zeller, R. A. & Carmines, E. G. (1980). *Measurement in the social sciences: The link between theory and data*. CUP Archive. [↑](#footnote-ref-1)
2. Haider, N., Yavlinsky, A., Chang, Y. M., Hasan, M. N., Benfield, C., Osman, A. Y., ... & Kock, R. (2020). The Global Health Security index and Joint External Evaluation score for health preparedness are not correlated with countries' COVID-19 detection response time and mortality outcome. *Epidemiology & Infection*, *148*. [↑](#footnote-ref-2)
3. Bauer P, Brugger J, König F, Posch M. An international comparison of age and sex dependency of COVID-19 deaths in 2020: a descriptive analysis. Sci Rep. 2021 Sep 27;11(1):19143. [↑](#footnote-ref-3)
4. Ioannidis, J. P., Axfors, C. & Contopoulos-Ioannidis, D. G. Second versus first wave of COVID-19 deaths: Shifts in age distribution and in nursing home fatalities. Environ Res. 2021 Apr;195:110856 [↑](#footnote-ref-4)
5. O’Driscoll, M., Dos Santos, G. R., Wang, L., Cummings, D. A., Azman, A. S., Paireau, J., ... & Salje, H. (2021). Age-specific mortality and immunity patterns of SARS-CoV-2. *Nature*, *590*(7844), 140-145. [↑](#footnote-ref-5)
6. <https://www.cdc.gov/coronavirus/2019-ncov/covid-data/investigations-discovery/hospitalization-death-by-age.html> [↑](#footnote-ref-6)
7. Cohen JF, Korevaar DA, Matczak S, Chalumeau M, Allali S, Toubiana J. COVID-19-Related Fatalities and Intensive-Care-Unit Admissions by Age Groups in Europe: A Meta-Analysis. Front Med (Lausanne). 2021 Jan 14;7:560685. [↑](#footnote-ref-7)
8. <https://www.statista.com/> [↑](#footnote-ref-8)
9. Cohen JF, Korevaar DA, Matczak S, Chalumeau M, Allali S, Toubiana J. COVID-19-Related Fatalities and Intensive-Care-Unit Admissions by Age Groups in Europe: A Meta-Analysis. Front Med (Lausanne). 2021 Jan 14;7:560685. [↑](#footnote-ref-9)
10. Mallapaty, S. (2020). The coronavirus is most deadly if you are old and male. *Nature*, *585*(7823), 16-17. [↑](#footnote-ref-10)
11. Younsi, F. Z. (2017). Multicriteria decision making with ELECTRE III, SOLAP and GIS for spatiotemporal tuberculosis analytics. *Medical Technologies Journal*, *1*(3), 57-58. [↑](#footnote-ref-11)
12. de Paulaa, N. O. B., de Araújo Costab, I. P., Drumonda, P., Moreirab, M. Â. L., Gomesb, C. F. S., & dos Santosa, M. (2021). Strategic support for the distribution of vaccines against Covid-19 to Brazilian remote areas: A multicriteria approach in the light of the ELECTRE-MOr method. Procedia Computer Science [↑](#footnote-ref-12)
13. de Araújo Costa, I. P., Sanseverino, A. M., dos Santos Barcelos, M. R., Belderrain, M. C. N., Gomes, C. F. S., & dos Santos, M. (2021). Choosing flying hospitals in the fight against the COVID-19 pandemic: structuring and modeling a complex problem using the VFT and ELECTRE-MOr methods. *IEEE Latin America Transactions*, *19*(6), 1099-1106. [↑](#footnote-ref-13)
14. Farid, F., & Donyatalab, Y. (2021, August). Optimal Selecting of Sanitarium Sites for COVID-19 Patients in Iran by Applying an Integrated ELECTRE-VIKOR Method in q-ROFSs Environment. In *International Conference on Intelligent and Fuzzy Systems* (pp. 541-551). Springer, Cham. [↑](#footnote-ref-14)
15. references [69-75] in the paper, and Anić, I., Ćirović, G., & Šormaz, G. The classification of commercial property using ELECTRE multi-criteria decision making methods. *Operational research and quantitative methods in management*, 1233. [↑](#footnote-ref-15)
16. <http://blueair.sf.bg.ac.rs/index2.php> (link BEMLO benchmarking network, Benchmarking tool introduced within Interreg project BlueAir). [↑](#footnote-ref-16)
17. OECD, & European Union. (2020). Health at a Glance: Europe 2020: State of Health in the EU Cycle. [↑](#footnote-ref-17)
18. Anderson RM, Heesterbeek H, Klinkenberg D, Hollingsworth TD. How will country-based mitigation measures influence the course of the COVID-19 epidemic? The Lancet. 2020 Mar 21;395(10228):931–4. pmid:32164834 [↑](#footnote-ref-18)
19. Jamison, D. T., & Wu, K. B. (2021). The East–West divide in response to COVID-19. *Engineering*, *7*(7), 936-947. [↑](#footnote-ref-19)
20. Arjen Boin , Martin Lodge & Marte Luesink (2020): Learning from the
    COVID-19 crisis: an initial analysis of national responses, Policy Design and Practice, DOI:10.1080/25741292.2020.1823670 [↑](#footnote-ref-20)
21. Toshkov, D., Carroll, B., & Yesilkagit, K. (2022). Government capacity, societal trust or party preferences: what accounts for the variety of national policy responses to the COVID-19 pandemic in Europe?. *Journal of European Public Policy*, *29*(7), 1009-1028. [↑](#footnote-ref-21)
22. Devine, D., Gaskell, J., Jennings, W., & Stoker, G. (2021). Trust and the coronavirus pandemic: What are the consequences of and for trust? An early review of the literature. *Political Studies Review*, *19*(2), 274-285. [↑](#footnote-ref-22)
23. Ylli, A., Wu, Y. Y., Burazeri, G., Pirkle, C., & Sentell, T. (2020). The lower COVID-19 related mortality and incidence rates in Eastern European countries are associated with delayed start of community circulation. *PLoS One*, *15*(12), e0243411. [↑](#footnote-ref-23)
24. Greer, S. L., King, E. J., da Fonseca, E. M., & Peralta-Santos, A. (2020). The comparative politics of COVID-19: The need to understand government responses. *Global public health*, *15*(9), 1413-1416. [↑](#footnote-ref-24)
25. The Guardian (May 5^th^, 2020). “Why has eastern Europe suffered less from coronavirus than the west?”, retrieved from <https://www.theguardian.com/world/2020/may/05/why-has-eastern-europe-suffered-less-from-coronavirus-than-the-west> [↑](#footnote-ref-25)
26. Guasti, P. (2020). The impact of the Covid-19 pandemic in Central and Eastern Europe: The rise of autocracy and democratic resilience. *Democratic theory*, *7*(2), 47-60. [↑](#footnote-ref-26)
27. Rudan I. A cascade of causes that led to the COVID-19 tragedy in Italy and in other European Union countries. J Glob Health. 2020 Jun;10(1):010335. [↑](#footnote-ref-27)
28. Soriano V, Barreiro P. Why such excess of mortality for COVID-19 in Spain? Ther Adv Infect Dis. 2020 Jun 4; 7:2049936120932755. [↑](#footnote-ref-28)
29. Konstantinoudis G, Cameletti M, Gómez-Rubio V, Gómez IL, Pirani M, Baio G, Larrauri A, Riou J, Egger M, Vineis P, Blangiardo M. Regional excess mortality during the 2020 COVID-19 pandemic in five European countries. Nat Commun. 2022 Jan 25;13(1):482. [↑](#footnote-ref-29)
30. Zipursky, J. S., & Redelmeier, D. A. (2020). Mobility and mortality during the COVID-19 pandemic. *Journal of General Internal Medicine*, *35*(10), 3100-3101. [↑](#footnote-ref-30)
31. Filonchyk, M., Hurynovich, V., & Yan, H. (2021). Impact of Covid-19 lockdown on air quality in the Poland, Eastern Europe. *Environmental Research*, *198*, 110454. [↑](#footnote-ref-31)
32. Menut, L., Bessagnet, B., Siour, G., Mailler, S., Pennel, R., & Cholakian, A. (2020). Impact of lockdown measures to combat Covid-19 on air quality over western Europe. *Science of the Total Environment*, *741*, 140426. [↑](#footnote-ref-32)
